# Seasonal consumption of polyphenol-rich fruits affects the hypothalamic leptin signaling system in a photoperiod-dependent mode

**DOI:** 10.1038/s41598-018-31855-y

**Published:** 2018-09-11

**Authors:** Maria Ibars, Gerard Aragonès, Andrea Ardid-Ruiz, Albert Gibert-Ramos, Anna Arola-Arnal, Manuel Suárez, Cinta Bladé

**Affiliations:** Universitat Rovira i Virgili, Department of Biochemistry and Biotechnology, Nutrigenomics Research Group, Tarragona, Spain

## Abstract

Leptin has a central role in the maintenance of energy homeostasis, and its sensitivity is influenced by both the photoperiod and dietary polyphenols. The aim of this study was to investigate the effect of seasonal consumption of polyphenol-rich fruits on the hypothalamic leptin signaling system in non-obese and obese animals placed under different photoperiods. Non-obese and diet-induced obese male Fischer 344 rats were placed under either a short-day (SD) or long-day (LD) photoperiod and were supplemented with either 100 mg/kg of lyophilized red grapes or cherries. In non-obese animals, both fruits reduced energy balance independent of the photoperiod to which they were placed. However, the hypothalamic gene expression of *Pomc* was significantly up-regulated only in the SD photoperiod. In contrast, in obese animals only cherry significantly decreased the energy balance, although both fruits were able to counteract the diet-induced increase in hypothalamic *AgRP* mRNA levels when consumed during the SD photoperiod. In conclusion, the consumption of rich-polyphenol fruits may increase leptin sensitivity through the modulation of the hypothalamic leptin signal pathway mainly when consumed in the SD photoperiod. Therefore, fruit seasonality should be considered, as it can influence energy homeostasis and obesity.

## Introduction

Leptin is a hormone produced by adipose tissue that has a key role in the central regulation of energy homeostasis^1^. The main target of leptin is the hypothalamic arcuate nucleus (ARC), where it activates proopiomelanocortin (POMC) anorexigenic neurons and inhibits agouti related peptide (AgRP) and neuropeptide Y (NPY) orexigenic neurons. Consequently, it modulates the second-order neuron activity of both melanocortin 4 receptor (MC4R)^2^ and NPY1 receptor (NPY1R)^3^. As a result, leptin produces a satiating effect and an increase in energy expenditure to maintain body weight^4^. Remarkably, leptin secretion follows circadian^5,6^ and circannual^7^ rhythms. Various studies with mammals sensitive to photoperiods have shown that animals develop adaptive leptin resistance in long-day periods to overcome periods of food scarcity^8^. This means that, despite presenting high leptin levels in blood, leptin is unable to produce anorexigenic effects, providing animals with sufficient energy stores for future short-day seasons. This peculiar metabolic behavior described in mammals has been recently considered an evolutionary mechanism for survival^9^.

Westernized society has developed inappropriate dietary patterns that contribute to the obesity epidemic, and fruit consumption is advised because fruits are a valuable source of nutrients with health-promoting properties, which supports their daily consumption. Furthermore, fruit contains phytochemicals that, while not essential for life, can exert long-term beneficial effects. Among them, polyphenols are an important group of compounds present in fruits^10^, and some studies have recently demonstrated that specific polyphenols increase leptin sensitivity in obese animals^11–13^.

In the modern era, a broad range of fruits is commonly available, and people can choose to consume seasonal and/or out-of-season fruits. However, the effects of consumption of fruits that are in-season or out-of-season on leptin sensitivity have not yet been studied. Our goal was to mimic seasonality by adapting animals to different photoperiods —short-day (SD) photoperiods for autumn and long-day (LD) photoperiod for spring— to investigate the effects of seasonal fruit consumption on the hypothalamic leptin signaling system. Given the role of leptin in maintaining body weight, this study was performed in both obese and non-obese animals. Additionally, numerous studies report the metabolic protective effects of grapes, grape by-products, cherries or their pure compounds^14–19^, and thus, we chose red grape and cherry as representative polyphenol-rich fruits for autumn and spring, respectively.

Our approach might provide valuable information to design strategies of fruit consumption to counteract positive energy balance through modulation of the hypothalamic leptin signaling pathway and its downstream effectors.

## Results

### Fruit consumption modulated energy balance by altering energy expenditure and food intake in a photoperiod-dependent manner in non-obese animals

**Figure 1:**
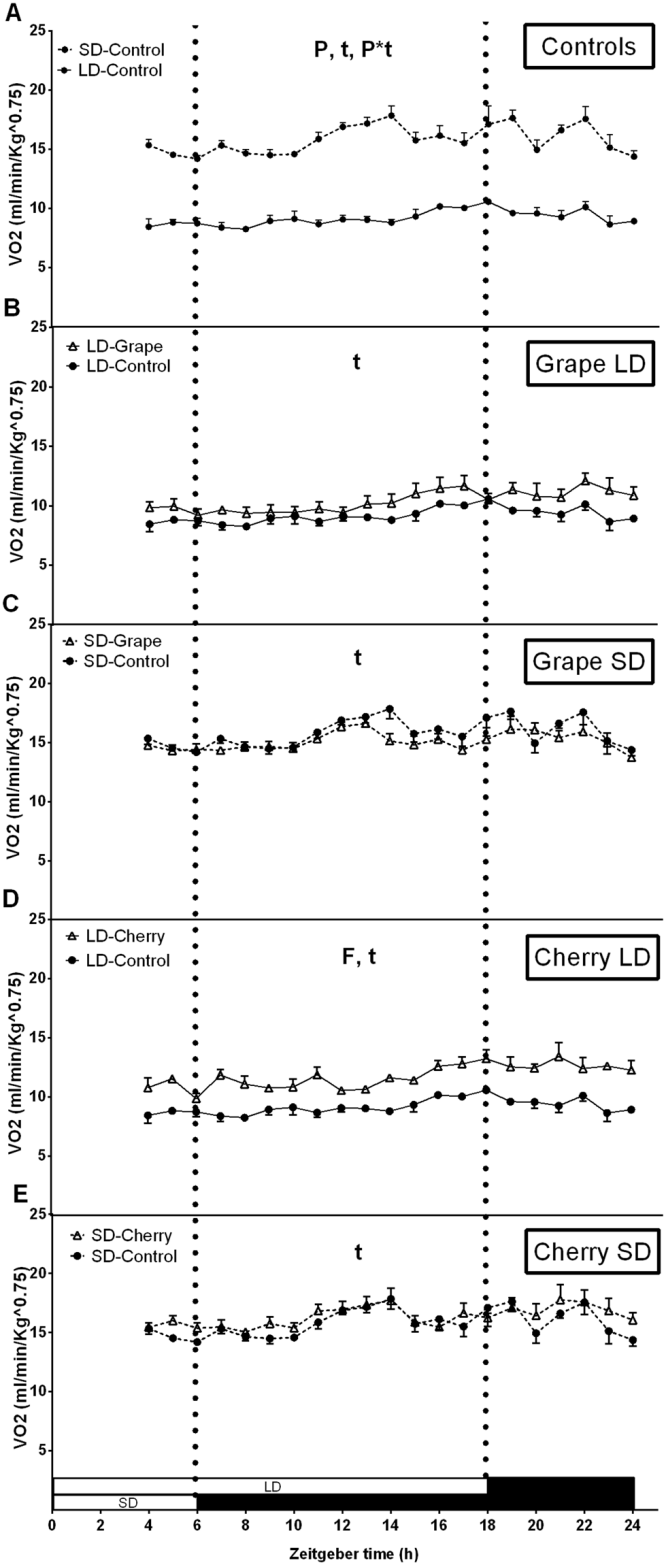
Effect of photoperiod and seasonal fruit supplementation on oxygen consumption (VO_2_) in non-obese animals. Twenty hours of indirect calorimetry were measured in animals fed standard chow diet + vehicle (**A**) or 100 mg/kg of lyophilized grapes (**B**,**C**) or cherries (**D**,**E**) for a 10-week-period and adapted to long-day (LD) or short-day (SD) photoperiod. Values are mean ± SE (n = 6). P, photoperiod effect; F, fruit effect; F*t, interaction of fruit treatment and time; P*t, interaction of photoperiod and time, assessed by two-way ANOVA (*P* < 0.05).

Table 1 shows the effect of the photoperiod and fruit consumption on body weight, total fat mass, cumulative energy intake, 24 h energy expenditure, energy balance and RQ values in non-obese animals. Supplementing animals with 100 mg/kg of grape or cherry for 10 weeks did not produce significant changes in body weight in either of the two experimental photoperiods assessed in this study, and the photoperiod itself did not significantly affect body weight. However, control animals placed in the SD photoperiod showed significantly lower fat mass percentage than those placed in the LD photoperiod, and grape consumption completely abolished this photoperiod-dependent effect. Moreover, the photoperiod itself and cherry consumption did not significantly alter food intake values, and only the consumption of grapes significantly reduced it in the SD photoperiod. Animals placed in the SD photoperiod significantly increased VO_2_ values, indicating that non-obese animals expended more energy in the SD photoperiod than in the LD photoperiod (Fig. 1A). Interestingly, grape consumption did not alter this pattern (Fig. 1B,C), but cherry significantly increased VO_2_ values of animals placed in the LD photoperiod (Fig. 1D,E).

**Table 1:** Effect of photoperiod and fruit consumption on body weight gain, fat mass, cumulative food intake, energy expenditure, energy balance and respiratory quotient in non-obese animals.

	Photoperiod	Control	Grape	ANOVA^b^	Cherry	ANOVA^b^
Body weight gain (g)	LD	89.17 ± 4.1	91.33 ± 4.7	*ns*	83.80 ± 5.0	*ns*
	SD	84.17 ± 4.8	80.67 ± 1.7		90.00 ± 7.0	
Fat mass (g)	LD	55.64 ± 4.41	54.36 ± 4.71	*ns*	58.38 ± 3.11	*P*
	SD	45.06 ± 1.29	51.18 ± 3.12		50.41 ± 2.82	
Fat mass (%)	LD	14.38 ± 0.7	14.40 ± 1.2	*ns*	15.17 ± 0.7	*P*
	SD	12.52 ± 0.3	14.98 ± 0.3		13.43 ± 0.6	
Cumulative food intake (MJ)	LD	2.26 ± 0.1	2.20 ± 0.0	*F*	2.21 ± 0.0	*ns*
	SD	2.26 ± 0.0	2.14 ± 0.0		2.23 ± 0.1	
24 h energy expenditure (MJ/Kg^0.75^)	LD	0.13 ± 0.00	0.14 ± 0.01	P, F*P	0.16 ± 0.00	*P, F*
	SD	0.21 ± 0.01	0.20 ± 0.00*		0.22 ± 0.01	
24 h energy balance^a^ (MJ)	LD	0.10 ± 0.0	0.09 ± 0.0	*P, F*	0.07 ± 0.01	*P, F*
	SD	0.04 ± 0.0	0.01 ± 0.0		0.00 ± 0.01	
24 h RQ	LD	0.86 ± 0.0	0.86 ± 0.0	*P*	0.83 ± 0.01	*P*
	SD	0.80 ± 0.0	0.82 ± 0.0		0.78 ± 0.01	

LD, long day; ns, non-significant; RQ, respiratory quotient; SD, short day. Values are presented as the mean ± SE of six animals per group. ^a^Energy balance was estimated by difference between energy intake and energy expenditure. ^b^Denotes two-way ANOVA analysis: *P*, photoperiod effect; *F*, fruit effect (*P* ≤ 0.05). *Effect of fruit on photoperiod group determined by Student’s *t* test (*P* ≤ 0.05).

Moreover, fruit consumption reduced 24 hour energy balance in both photoperiods, indicating that the energy expenditure was increased compared to the energy intake. This effect was significantly enhanced by grape and cherry consumption in SD photoperiod suggesting that the SD photoperiod and fruit consumption directed energy homeostasis towards a zero balance. Additionally, animals placed in SD presented a significant reduction in RQ values, indicating that LD photoperiod favored carbohydrate utilization, whereas SD photoperiod favored lipid use.

Together, these results indicate that the SD photoperiod, compared to the LD photoperiod, increased energy expenditure and favored lipid use as energetic substrate in non-obese animals, resulting in reduced energy balance and body fat mass percentage. Remarkably, cherry and grape consumption enhanced the decrease on energy balance.

### Fruit consumption up-regulated hypothalamic gene expression of Pomc in SD photoperiod, which was associated with lower leptin levels in non-obese animals

**Figure 2:**
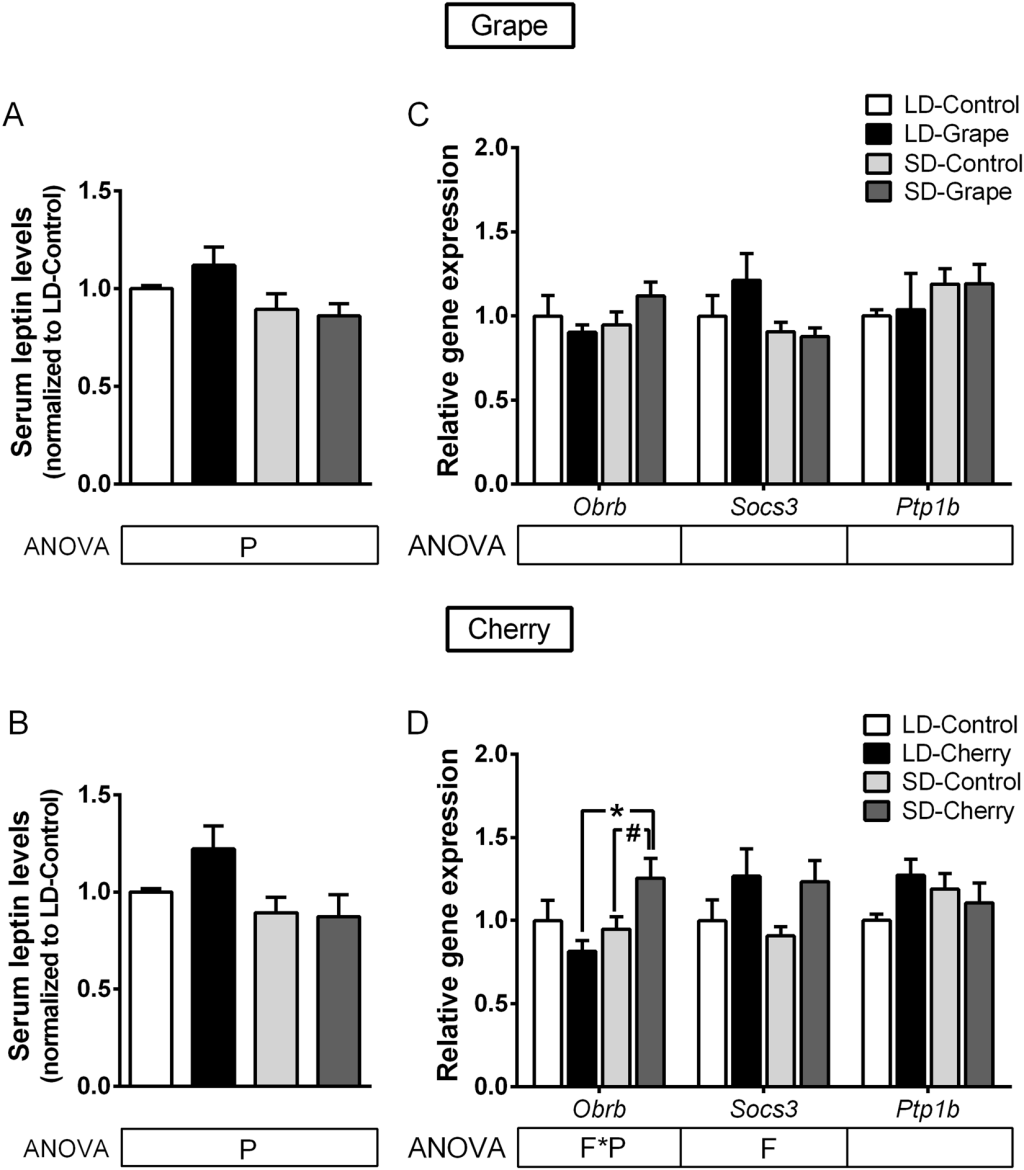
Effect of photoperiod and seasonal fruit supplementation on hypothalamic leptin sensitivity in non-obese animals. (**A**,**B**) Serum leptin concentrations were measured in animals fed standard chow diet + vehicle or 100 mg/kg of lyophilized grapes or cherries for a 10-week-period and submitted to long-day (LD) or short-day (SD) photoperiods. (**C**,**D**) Gene expression of long-form leptin receptor (*Obrb*) and negative regulator molecules *Socs3* and *Ptp1b* were analyzed by quantitative PCR. Values were normalized against LD-vehicle group for leptin concentration and gene expression. Data represent mean ± SE (n = 6). P, photoperiod effect; F, fruit effect; F*P, interaction of photoperiod and fruit treatment assessed by two-way ANOVA (*P* < 0.05). *Effect of photoperiod in fruit-treated groups; ^#^Effect of fruit in photoperiod group determined by Student’s *t* test (*P* < 0.05).

Thus, the next goal was to investigate whether photoperiod and/or fruit consumption could modulate leptin system in these animals. The photoperiod significantly affected serum concentrations of leptin, increasing leptin levels in the LD photoperiod (Fig. 2A,B). In addition, fruit consumption enhanced them in the LD photoperiod compared to the SD photoperiod. Since leptin regulates body fat mass, food intake and energy expenditure, we further analyzed the correlation between these parameters in each photoperiod (Supplementary Fig. S1). Interestingly, leptin levels showed a positive relationship with 24 h energy expenditure in SD photoperiod (*r* = 0.335), but it did not reach statistical significance.

**Figure 3:**
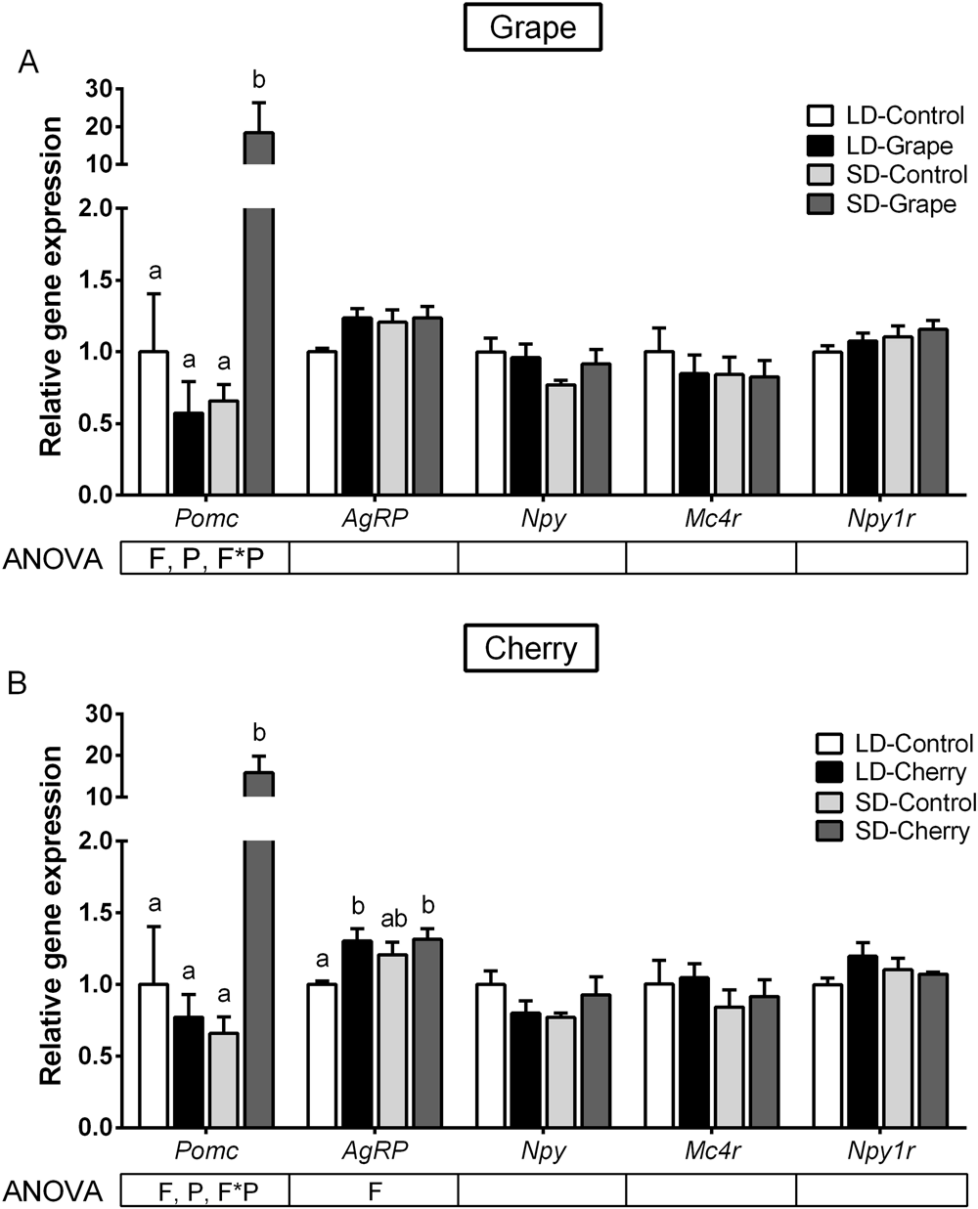
Effect of photoperiod and seasonal fruit supplementation on hypothalamic neuropeptides regulated by leptin in non-obese animals. Hypothalamic gene expression of neuropeptides evaluated by quantitative PCR on animals fed standard chow diet + vehicle or 100 mg/kg of lyophilized grapes (**A**) or cherries (**B**) for 10-week-period and submitted to long-day (LD) or short-day (SD) photoperiods. Values were normalized against LD-vehicle group. Data represent mean ± SE (n = 6). P, photoperiod effect; F, fruit effect; F*P, and interaction of photoperiod and fruit treatment assessed by two-way ANOVA (*P* ≤ 0.05). Different letters denote significant changes assessed by one-way ANOVA and Tukey’s post hoc test (*P* < 0.05).

Hypothalamic leptin signaling was assessed by analyzing the gene expression of leptin receptor isoform B (*Obrb*) and the cellular regulators *Socs3* and *Ptp1b*. Accordingly, mRNA levels of *Obrb*, *Socs3* and *Ptp1b* were not modified by the photoperiod or grape consumption (Fig. 2C). However, cherry significantly affected *Obrb* expression in a way that was dependent on the photoperiod, up-regulating and down-regulating its gene expression in the SD photoperiod and LD photoperiod, respectively (Fig. 2D). Cherry consumption also resulted in significant overexpression of *Socs3* mRNA levels in both photoperiods. We next investigated the hypothalamic mRNA levels of *Pomc*, *Agrp* and *Npy* neuropeptides, all of which were produced by first-order neurons in the ARC under leptin regulation. In addition, the mRNA levels of *Mc4r* and *Npy1r* receptors were also examined as the main targets of POMC, AgRP and NPY in second-order neurons. Notably, grape and cherry intake increased *Pomc* expression (20 times) exclusively in the SD photoperiod (Fig. 3A,B), indicating that the effect of grape and cherry was clearly dependent on the photoperiod in which they were consumed. In addition, cherry consumption significantly increased *Agrp* expression in both photoperiods, however to a lesser extent (Fig. 3B). The photoperiod or fruit consumption did not modify the expression of *Npy*, *Mc4r* and *Npy1r*.

### Fruit consumption modulated energy balance by altering food intake and RQ values in a photoperiod-dependent manner in obese animals

**Figure 4:**
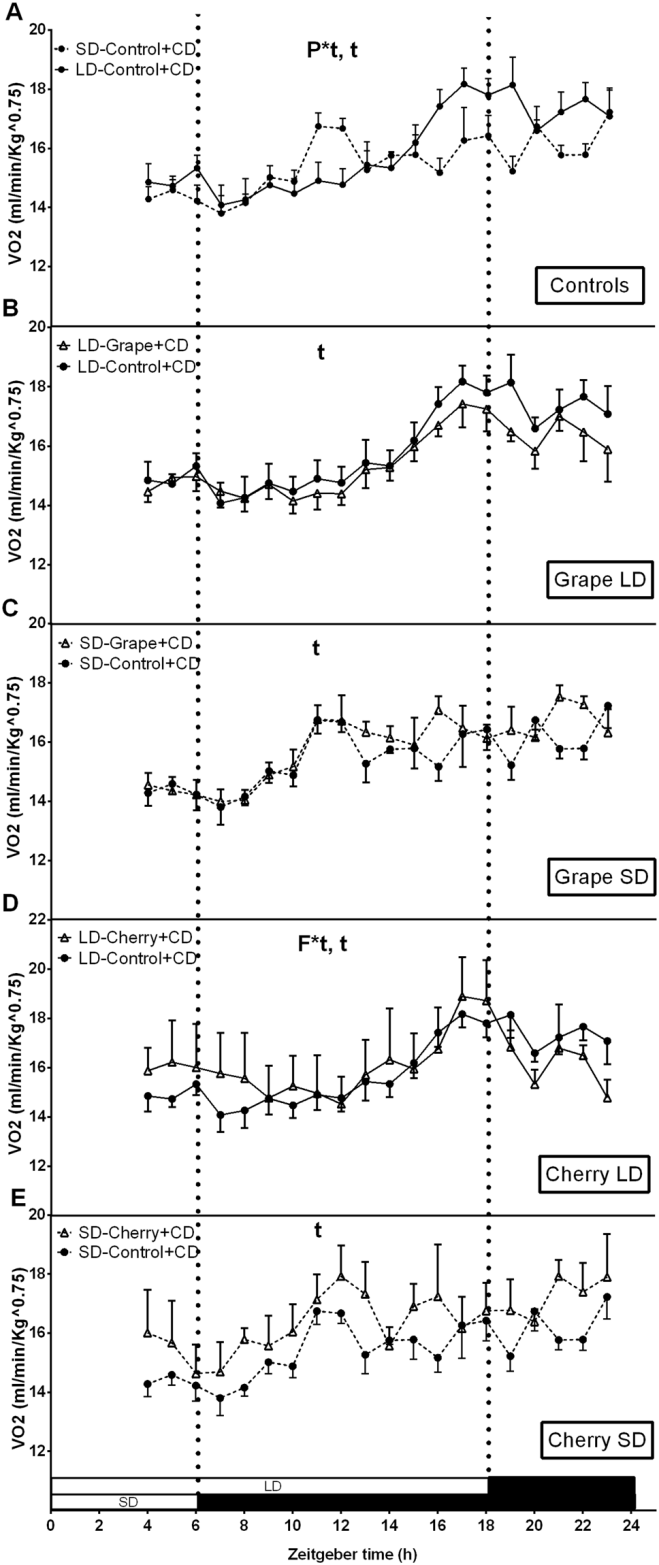
Effect of photoperiod and seasonal fruit supplementation on oxygen consumption (VO_2_) in obese animals. Twenty hours of indirect calorimetry were measured in animals fed cafeteria diet + vehicle (**A**) or 100 mg/kg of lyophilized grapes (**B**,**C**) or cherries (**D**,**E**) for a 7-week-period and submitted to long-day (LD) or short-day (SD) photoperiods. Values are mean ± SE (n = 6). F*t, interaction of fruit treatment and time; P*t, interaction of photoperiod and time, assessed by two-way ANOVA (*P* < 0.05).

The observation that the photoperiod and fruit consumption conditioned fat mass percentage, energy balance and hypothalamic leptin system in non-obese animals prompted us to investigate how fruit consumption and photoperiod could affect energy homeostasis in obese animals that presented impaired leptin sensitivity. Rats were fed a cafeteria diet for 7 weeks, together with an oral dose of 100 mg/kg of lyophilized grape or cherry. As depicted in Table 2, body weight and total body fat accumulation were not affected by photoperiod or fruit consumption. However, these animals displayed significantly lower cumulative energy intake in the SD photoperiod compared to that in the LD photoperiod. In addition, cherry consumption reduced the cumulative energy intake when consumed in the LD photoperiod, whereas no effect was observed after grape consumption. The photoperiod or fruit consumption did not significantly alter 24-hour energy expenditure (Table 2 and Fig. 4).

**Table 2:** Effect of photoperiod and fruit consumption on body weight gain, fat mass, cumulative food intake, energy expenditure, energy balance and respiratory quotient in obese animals.

	Photoperiod	Control	Grape	ANOVA^b^	Cherry	ANOVA^b^
Body weight gain (g)	LD	113.31 ± 5.4	128.36 ± 4.3	*ns*	121.09 ± 3.2	*ns*
	SD	116.74 ± 6.6	117.39 ± 4.3		119.70 ± 5.9	
Fat mass (g)	LD	89.51 ± 3.80	96.49 ± 2.90	*ns*	86.56 ± 2.23	*ns*
	SD	85.84 ± 3.27	87.29 ± 5.48		88.29 ± 5.31	
Fat mass (%)	LD	22.03 ± 0.6	22.57 ± 0.6	*ns*	21.70 ± 0.7	*ns*
	SD	21.53 ± 0.8	21.10 ± 1.3		21.82 ± 1.0	
Cumulative food intake (MJ)	LD	5.79 ± 0.2	5.72 ± 0.2	*P*	5.13 ± 0.2	*F*
	SD	5.43 ± 0.2	5.20 ± 0.1		5.01 ± 0.1	
24 h energy expenditure (MJ/Kg^0.75^)	LD	0.23 ± 0.01	0.23 ± 0.01	*ns*	0.22 ± 0.01	*ns*
	SD	0.23 ± 0.01	0.24 ± 0.00		0.23 ± 0.01	
24 h energy balance^a^ (MJ)	LD	0.83 ± 0.1	0.79 ± 0.0	*P*	0.40 ± 0.1*	F, F*P
	SD	0.60 ± 0.1	0.46 ± 0.1		0.55 ± 0.1	
24 h RQ	LD	0.78 ± 0.0	0.78 ± 0.0	*P*	0.79 ± 0.0	F*P
	SD	0.81 ± 0.0	0.80 ± 0.0		0.78 ± 0.0*	

LD, long day; ns, non-significant; RQ, respiratory quotient; SD, short day. Values are presented as the mean ± SEM of ten animals per group. ^a^Energy balance was estimated by difference between energy intake and energy expenditure. ^b^Denotes two-way ANOVA analysis: *P*, photoperiod effect; *F*, fruit effect; F*P, interaction effect of photoperiod and fruit treatment (*P* ≤ 0.05). *Effect of fruit on photoperiod group determined by Student’s *t* test (*P* ≤ 0.05).

Notably, a positive 24-hour energy balance was observed in all experimental conditions, except for those placed in the SD photoperiod. Cherry significantly reduced 24-hours energy balance when consumed in the LD photoperiod, indicating that the lower 24 h-energy balance exhibited by obese animals in this photoperiod was a direct consequence of the reduced energy intake. These data totally differ from those obtained in non-obese animals, in which cherry reduced energy balance in both photoperiods and in the case of animals placed on LD that was due to an increase in 24-hour energy expenditure.

Finally, the photoperiod also modulated RQ values in obese animals. Specifically, these animals presented a significant increase in RQ values in the SD photoperiod, indicating that the LD photoperiod favored lipid use as energetic substrates whereas the SD photoperiod favored carbohydrates. In addition, cherry consumption in the SD photoperiod significantly decreased RQ values in obese animals.

### Photoperiod and fruit consumption modulated hypothalamic *Socs3* and Agpr gene expression but not leptin levels in obese animals

**Figure 5:**
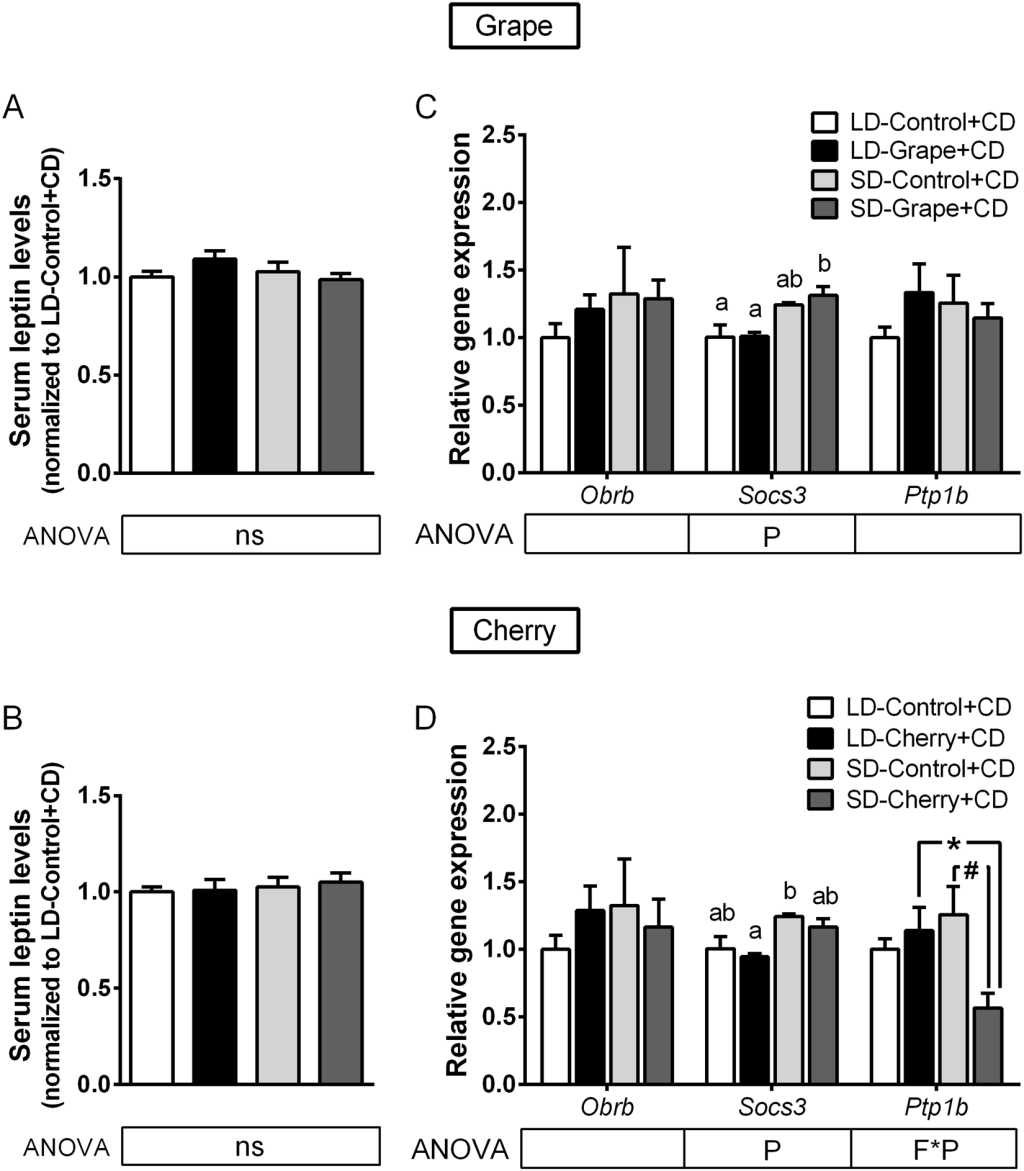
Effect of photoperiod and seasonal fruit consumption on hypothalamic leptin sensitivity in obese animals. (**A**,**B**) Serum leptin was measured in animals fed a cafeteria diet (CD) + vehicle or 100 mg/kg of lyophilized grapes or cherries for 7-week-period and submitted to long-day (LD) or short-day (SD) light cycle. (**C**,**D**) Gene expression of long form of leptin receptor (*Obrb*) and negative regulator molecules *Socs3* and *Ptp1b* were analyzed. Values were normalized against LD-vehicle group for leptin concentration and gene expression. Data represent mean ± SE (n = 6). P, photoperiod effect; F, fruit effect; F*P, interaction of photoperiod and fruit treatment assessed by two-way ANOVA (*P* < 0.05). Different letters denote significant changes assessed by one-way ANOVA and Tukey’s post hoc test (*P* < 0.05). *Effect of photoperiod in fruit treated groups; ^#^Effect of fruit in photoperiod group determined by Student’s t test (*P* < 0.05).

The circulating levels of leptin were similar in all three groups of animals (Fig. 5A,B), indicating that feeding animals with an obesogenic diet completely disrupted the photoperiod-dependent profile of circulating leptin levels observed in non-obese animals. Similarly, the correlation observed between leptin levels and 24 h energy expenditure in SD photoperiod in non-obese animals were also abolished in diet-induced obesity (Supplementary Fig. S2).

We next studied whether the photoperiod and/or fruit consumption directly modulated hypothalamic leptin signaling in obesity by determining the gene expression of *Obrb*, *Socs3* and *Ptp1b*. Among them, only *Socs3* mRNA levels were sensitive to the photoperiod, increasing its values in the SD photoperiod (Fig. 5C,D). Interestingly, grape consumption enhanced the gene expression of *Socs3* in the SD photoperiod, whereas cherry consumption repressed both *Socs3* and *Ptp1b* gene expression when consumed in the SD photoperiod, indicating that the expression of genes involved in the regulation of leptin signaling was more sensitive to modulation by the photoperiod and fruit consumption in obese animals than in non-obese ones.

**Figure 6:**
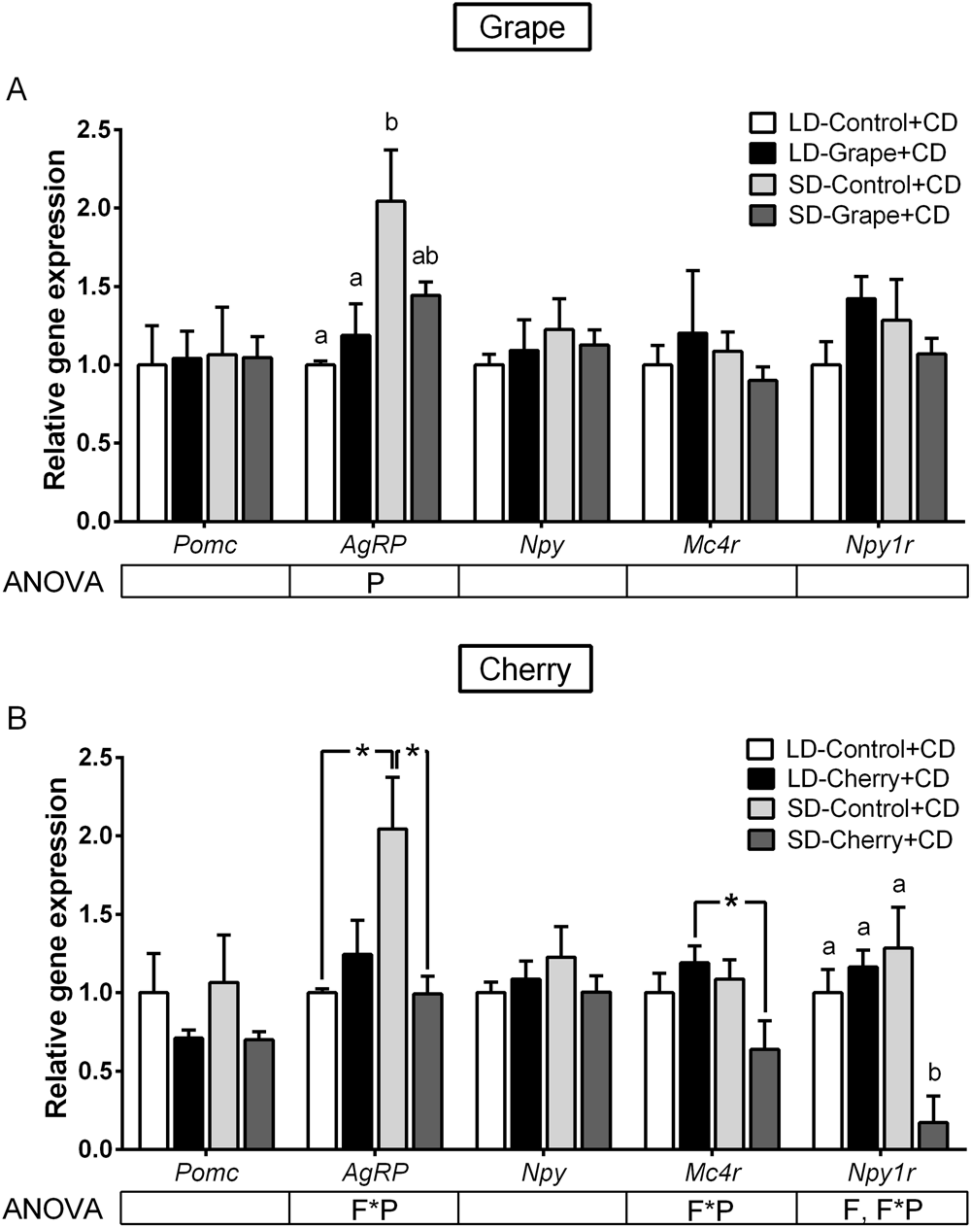
Effect of photoperiod and seasonal fruit supplementation on hypothalamic neuropeptides regulated by leptin in obese animals. Hypothalamic gene expression of neuropeptides was evaluated by qPCR on animals fed cafeteria diet (CD) + vehicle or 100 mg/kg of lyophilized grapes (**A**) or cherries (**B**) for a 7-week-period and submitted to long-day (LD) or short-day (SD) light cycle. Values were normalized against LD-Control group. Data represent mean ± SE (n = 6). P, photoperiod effect; F, fruit effect; F*P, interaction of photoperiod and fruit treatment assessed by two-way ANOVA (*P* < 0.05). Different letters denote significant changes assessed by one-way ANOVA and Tukey post hoc test (*P* < 0.05). *Effect of photoperiod in fruit-treated groups; ^#^Effect of fruit in photoperiod groups determined by Student’s t test (*P* < 0.05).

We also investigated the hypothalamic mRNA levels of *Pomc*, *Agrp*, *Npy*, *Mc4r* and *Npy1r*. Obese animals placed in the SD photoperiod presented a marked overexpression of the orexigenic *Agrp* neuropeptide (Fig. 6A,B), and the consumption of both fruits in this photoperiod prevented the up-regulation of the gene. Additionally, cherry consumption also modulated the gene expression of *Mc4r* and *Npy1r* by repressing the genes when cherry was consumed in the SD photoperiod (Fig. 6B). These results indicate that in the SD photoperiod, hypothalamic *Socs3* and *Agrp* gene expression was up-regulated in obese animals compared to that in the LD photoperiod. In contrast, the consumption of grape or cherry strongly reversed this photoperiod-dependent gene expression pattern, repressing the overexpression of *Agrp* when fruits were consumed in the SD photoperiod.

## Discussion

Great attention has been paid to calorie consumption and diet composition to improve metabolic health and decrease obesity^20^. However, there is a lack of studies focusing on seasonal foods and their detrimental or beneficial effects when consumed in- or out-of-season. Seasonality plays an important role in all organisms to maintain life balance^21^. Here, we present the results of this novel approach, showing that seasonal fruits can modulate the leptin system depending on the photoperiod in which they are consumed, in both healthy and obese animals.

The Siberian hamster (*Phodopus sungorus*) is the most widely used animal model to study photoperiod responses^22^. However, in this study we opted for the Fischer 344 strain rat because rat metabolism is more similar to that of humans^23^, and this strain is sensitive to the photoperiod^24^.

Non-obese animals presented the characteristic phenotype described for Fischer 344 rats placed in the LD or SD photoperiods, displaying lower fat mass^25,26^ and leptin concentrations^25,27^ in the SD condition. However, these rats did not present a significant reduction in body weight described as typical for the SD photoperiod. Remarkably, animals placed for a long period of time in a SD photoperiod become refractory to light cycle conditions, shifting to a LD physiology in order to accomplish reproduction and preserve species survival^28^. Specifically, this time is considered to be 10 weeks for Fischer 344 rats^29^, and rats in our experiment were kept 14 weeks at SD; thus, they could be in a refractory state. However, we selected 4 weeks of adaptation to the photoperiod plus 10 weeks of fruit treatment because we aimed to determine the long-term effect of seasonal fruit consumption in animals already adapted to a specific photoperiod.

The fat mass percentage reduction observed in non-obese rats placed at SD was associated with a lower energy balance (close to zero), which can be ascribed to increased energy expenditure rather than reduced food intake, as described by other authors^25,29^. Leptin is essential in the regulation of central energy homeostasis^30^. However, we did not observe any change in the expression of neuropeptides regulated by leptin or their receptors in second-order of non-obese animals at SD. Thus, other hormones, such as prolactin^31^, or an increase in leptin sensitivity^32,33^, induced by other leptin signaling components not determined in this study, could participate in this higher energy expenditure observed in SD. In this sense, small rodents, such as hamsters or field voles (*Microtus agrestis*), develop leptin resistance in LD due to decreased pSTAT3^33^, which was not evaluated in the present study.

Our objective was not to directly compare non-obese versus obese animals but rather to describe the fruit and photoperiod effects in the two models separately. However, it is important to note that the response of obese animals to photoperiod diverged significantly from that of lean animals. Only few studies have focused on the photoperiod effects to animals fed an obesogenic diet. However, a study using Fischer 344 rats fed a high-fat diet for 4 weeks^25^ agrees with our findings for both the loss of photoperiod regulation of fat mass and the photoperiod regulation of food intake, decreasing it in the SD condition. Thus, obese rats in the present study reduced their energy balance at SD as a consequence of reduced cumulative energy intake, without any modification to energy expenditure. In addition, the photoperiod effect on serum leptin levels was blunted when animals were on the obesogenic diet. However, leptin system was altered in SD, as indicated by a slight increase of *Socs3* and marked overexpression of *Agrp* mRNA levels in this photoperiod. Notably, it has been reported that leptin resistance associated with LD is caused by high serum leptin levels and *Socs3* overexpression^33^ whereas leptin sensitivity accompanying SD is associated with *Agrp* down-regulation in the arcuate nucleus^31^. Thus, the pattern of the leptin system in rats fed a cafeteria diet, together with the high serum leptin levels in SD, indicates that obese rats have lost the capacity to modulate leptin sensitivity according to photoperiods.

From all seasonal fruits, red grapes were selected as a representative fruit of autumn (SD) and cherry as a representative of fruit of spring (LD) because of their high content of polyphenols^34–36^. Red grapes contain mainly flavonoids, including anthocyanins, flavanols (monomers and proanthocyanidins), flavonols and non-flavonoids, such as phenolic acids and stilbenes (mainly resveratrol)^35,36^. Cherries also contain flavonoids, which include anthocyanins, flavanols (monomers and proanthocyanidins), and non-flavonoids such as phenolic acids (mainly hydroxycinnamates)^37,38^. Evidently, grape and cherry contain similar classes of polyphenols; cherries contain larger amounts of anthocyanins and hydroxycinnamic acids compared to grapes, but lack flavonols and stilbenes^39^. Notably, both fruits were given at a dose that in humans is equivalent to a small portion of fruit, below the serving size established in the European dietary guidelines^40^. Since fruits are a source of sugar, the rationale was that standard diet plus fruit supplementation could not exceed 10% of the total energy in the form of sugars, following the recommendations of the World Health Organization^41^.

The consumption of both fruits induced a lower energy balance in non-obese animals under both the SD and LD conditions, indicating that this metabolic effect was independent of the photoperiod condition. Remarkably, grape and cherry consumption reduced energy balance in different ways, for grape by reducing food intake but for cherry by increasing energy expenditure. The anorexigenic effect of grape consumption agrees with previous reported results demonstrating that grape seed proanthocyanidin extract reduces food intake in obese animals^11^. Interestingly, both cherry and grape intake resulted in overexpression of *Pomc* in the hypothalamus when consumed at SD. Thus, the enhanced anorexigenic effect of both fruits could account, at least partially, for the lower energy balance observed in rats consuming fruits at SD. However, *Pomc* was only modulated in SD, indicating the photoperiod-dependent ability of these fruits to modulate leptin signaling. In addition, cherry consumption also modulated hypothalamic *Obrb* expression in a photoperiod-dependent manner, increasing its expression in SD, fact that has been related to activation of POMC neurons and increased leptin sensitivity^42^. Thus, cherry clearly enhanced the out-of-season central leptin sensitivity in non-obese animals.

Remarkably, neither the energy balance reduction nor leptin sensitivity modulation induced by fruits were reflected in body weight or fat mass accretion. Thus, cherry and mainly grape should modulate other mechanisms that, in turn, counteract the expected fat mass reduction. In this sense, grape seed proanthocyanidins have adipogenic activity, overexpressing PPARγ, increasing adipocyte number and reducing adipocyte hypertrophy in visceral and subcutaneous fat pads^43^. Thus, grape can act at the adipocyte level, counteracting the expected response of white adipose tissue to a lower energy balance sate. In addition, grape seed proanthocyanidins increase mitochondrial oxidative capacity in skeletal muscle and brown adipose tissue^44,45^. Thus, the increase in energy expenditure in these organs could also contribute to the lower energy balance observed in rats fed grapes. There are no studies on the effects of cherry extracts on energy expenditure, but an effect of cherries on increasing energy expenditure by activating brown adipose tissue thermogenesis and/or the mitochondrial activity in other organs cannot be ruled out.

In obese rats, grape seed proanthocyanidins also increase hypothalamic leptin sensitivity^11^, associated with the overexpression of *Pomc*. In this case, no effects of grape intake were observed in obese animals in either photoperiod. However, grape consumption showed a tendency to decrease *Agrp* levels in SD without reaching significance. In contrast, cherries were very effective at modulating the leptin system in obese rats in a photoperiod-dependent mode. Specifically, cherry distinctly repressed the expression of *Agrp* and *Ptp1B* in SD. Interestingly, *Ptp1b* is a negative regulator of the leptin signaling pathway, and its inhibition increases leptin sensitivity in obese animals^45^. Furthermore, *Mc4r* and *Npy1r* were also repressed in SD, indicating that cherry was also effective in modulating the response in second-order neurons. Together, these results suggest that cherry intake improves central leptin sensitivity out-of-season in obese animals. This reduction in the orexigenic signal *Agrp* of cherry agrees with the decreased cumulative food intake in obese rats that consumed cherry in SD. In contrast, the photoperiod-dependent effect of cherry on hypothalamic leptin sensitivity completely differs from the lower energy balance and RQ values observed in obese rats consuming cherry at LD. Thus, as stated in non-obese animals, a cherry effect that increases energy expenditure in brown adipose tissue when it is consumed in season cannot be excluded.

The xenohormesis hypothesis^46^, as defined by Howitz and Sinclair, proposes that animal uses chemical signals from plants, mainly polyphenols, to determine the environmental status or food supply. This acknowledgment would allow animals to respond in advance to environmental alterations, thus increasing their probability of survival. Therefore, the specific polyphenol content in grape and cherry could act as a distinctive mark, informing rats of environmental conditions such as photoperiod. Reinforcing this idea, previous studies by our group have demonstrated that grape proanthocyanidins modulate circadian rhythms at the hypothalamic level^47^. Nevertheless, cherries are also rich on melatonin^48^, a hormone that provides information about day length, controlling seasonal phenotypic adjustments^49^. Interestingly, melatonin reduces the energy expenditure induced by cold exposure in the Siberian hamster placed at LD^50^ and stimulates *Pomc* expression in mice^51^. Therefore, the melatonin contained in cherries could significantly contribute to the metabolic effects induced by cherry intake observed in this study.

In summary, fruit consumption decreased the energy balance in non-obese and obese animals in a photoperiod-independent manner. Grape reduced cumulative food intake, whereas cherry increased energy expenditure. In contrast, both fruits modulated the leptin system in a photoperiod-dependent way, increasing leptin sensitivity in SD. In obese animals, only cherry intake modulated the leptin system, repressing *Agrp* and *Ptp1B* in SD. Thus, cherry intake modulated the central leptin system when consumed out-of-season in both obese animals and non-obese animals.

## Materials and Methods

### Fruit characteristics and preparation

Royal Down sweet cherries (*Prunus avium* L.) were purchased in Mercabarna (Barcelona, Spain). Black grapes (*Vitis vinifera* L.) of the Grenache variety were kindly provided by the producer (Tarragona, Spain). Cherry pits were removed, and grapes were kept intact. Fruits were frozen in liquid nitrogen and later ground. The homogenates were lyophilized in a Telstar LyoQuest lyophilizer (Thermo Fisher Scientific, Barcelona, Spain) at −55 °C. The nutritional and polyphenol composition of both fruits has been widely characterized and can be found as in Supplementary Tables S1–S4.

### Animal handling

The study was approved by the Animal Ethics Committee of University Rovira i Virgili (reference number 4249) and performed according to ethical standards in the European Directive 2010/62/EU for animal experiments. Thirty-six (n = 36) male Fischer 344 rats, 8 weeks of age and with 186 ± 17 g body weight, were purchased from Charles River Laboratories (Barcelona, Spain). The animals were pair-housed and distributed in two different rooms according to photoperiod. They were fed a standard chow diet (Panlab 04, Barcelona, Spain) with water *ad libitum*. Photoperiod groups consisted of a LD (18:6 h light:dark cycle) and SD (6:18 h light:dark cycle) groups and kept at 22 °C. After an adaptation period of 4 weeks, the animals in each photoperiod were weight-matched and distributed into 3 subgroups of 6 animals, the control group, and 2 groups supplemented with an oral dose of 100 mg/kg (diluted in water) of grapes or cherries for 10 weeks. Additionally, sixty (n = 60) male Fischer 344 rats, 8 weeks of age and 216 ± 15 g body weight, were pair-housed and distributed into two photoperiod groups, LD and SD, and kept at 22 °C. These animals were fed a standard chow diet plus a cafeteria diet with water *ad libitum*. The cafeteria diet consisted of bacon, carrots, cookies, foie gras, muffins, cheese and milk with 22% sucrose (w/v). After an adaptation period of 4 weeks, the animals in each photoperiod were also weight-matched and distributed into 3 subgroups of 10 animals, the control group, and 2 groups supplemented with an oral dose of 100 mg/kg (diluted in water) of grapes or cherries for 7 weeks. In both experiments, treatment was administered at 09:00 am every day and the control groups were supplemented with the same volume of a sugar solution (10 mg fructose/10 mg glucose). Animals were sacrificed by decapitation at the start of the light cycle (lights on at 09:00 am). Serum was obtained after blood clotting and centrifugation (2000 × *g*, 15 min, 4 °C). The hypothalamus was dissected and frozen immediately in liquid nitrogen. Body weight and food intake were measured weekly.

### Body composition and metabolic analysis

In the last week of the study, animals were subjected to magnetic resonance imaging (MRI) using EchoMRI-700 (Echo Medical Systems, LLC., TX, USA) to determine lean and fat mass. Data is expressed as a percentage of total body weight. During the last week of treatment, animals underwent 24 h of indirect calorimetry analyses using an Oxylet Pro System (Panlab, Barcelona, Spain). The program Metabolism 2.1.02 (Panlab, Barcelona, Spain) automatically calculated respiratory quotient (RQ) and energy expenditure as according to the Weir equation^52^. A nitrogen excretion rate of 135 μg/kg/min was also assumed^53^. Leptin concentration in serum samples was measured using a rat-specific enzyme immunoassay kit (Millipore, Madrid, Spain), following the manufacturer’s protocol.

### Gene expression analyses

The hypothalamus was processed to extract total RNA using TRIzol LS Reagent (Thermo Fisher, Madrid, Spain) followed by an RNeasy Mini Kit (Qiagen, Barcelona, Spain). RNA quantity and purity were measured with a NanoDrop 1000 spectrophotometer (Thermo Scientific, Madrid, Spain). RNA quality was assessed on a denaturing agarose gel. Reverse transcription was performed to obtain cDNA using the High-Capacity Complementary DNA Reverse Transcription Kit (Thermo Fisher). Gene expression was analyzed by quantitative PCR using the iTaq Universal SYBR Green Supermix (Bio-Rad) in the ABI prism 7900HT real-time PCR system (Applied Biosystems) using primers obtained from Biomers.net (Ulm, Germany). The forward and reverse primers used in this study can be found as Supplementary Table S5. The relative expression of each gene was calculated referring to *Ppia* and *Rplp0* housekeeping genes and normalized to the control group. The ∆∆Ct method was used and corrected for primer efficiency^54^.

### Statistical analysis

The effect of fruit supplementation (F), photoperiod (P) or the interaction between the two variables (F*P) was evaluated by two-way ANOVA. If a main effect was significant, differences between groups were further assessed using one-way ANOVA with Tukey’s post hoc test. In the absence of a main effect but with a significant interaction between fruit and photoperiod, pairwise comparisons were calculated among photoperiod groups and fruit groups using Student’s *t* test. GraphPad Prism 6 (GraphPad Software, La Jolla, CA, USA) was used for all statistical analysis. The values are expressed as means ± SE. *P* < 0.05 was considered significant.

## Electronic supplementary material

Supplementary information
