# Supplementary material for: Seasonal consumption of polyphenol-rich fruits affects the hypothalamic leptin signaling system in a photoperiod-dependent mode
Source: Sci Rep. 2018 Sep 11;8:13572. doi: 10.1038/s41598-018-31855-y (PMC6133929; doi:10.1038/s41598-018-31855-y)
Supplement: Supplementary file 1 — Supplementary information [file 41598_2018_31855_MOESM1_ESM.pdf]

Seasonal consumption of polyphenol-rich fruits affects the hypothalamic leptin signaling system in a photoperiod-dependent mode

Maria Ibars, Gerard Aragonès, Andrea Ardid-Ruiz, Albert Gibert-Ramos, Anna Arola-Arnal, Manuel Suárez & Cinta Bladé

Supplementary Table S1.
Nutrients present in raw Sweet Cherry (*Prunus avium* L.)

Nutrient	Unit	Value per 100 g
**Proximates**		
Water	g	82.25
Energy	kcal	63
Energy	kJ	263
Protein	g	1.06
Total lipid (fat)	g	0.2
Ash	g	0.48
Carbohydrate, by difference	g	16.01
Fiber, total dietary	g	2.1
Sugars, total	g	12.82
Sucrose	g	0.15
Glucose (dextrose)	g	6.59
Fructose	g	5.37
Maltose	g	0.12
Galactose	g	0.59
**Minerals**		
Calcium, Ca	mg	13
Iron, Fe	mg	0.36
Magnesium, Mg	mg	11
Phosphorus, P	mg	21
Potassium, K	mg	222
Zinc, Zn	mg	0.07
Copper, Cu	mg	0.06
Manganese, Mn	mg	0.07
Fluoride, F	µg	2
**Vitamins**		
Vitamin C, total ascorbic acid	mg	7
Thiamin	mg	0.027
Riboflavin	mg	0.033
Niacin	mg	0.154
Pantothenic acid	mg	0.199
Vitamin B-6	mg	0.049
Folate, total	µg	4

Folate, food	µg	4
Folate, DFE	µg	4
Choline, total	mg	6.1
Vitamin A	µg	3
Carotene, beta	µg	38
Lutein + zeaxanthin	µg	85
Vitamin E	mg	0.07
Tocopherol, beta	mg	0.01
Tocopherol, gamma	mg	0.04
Vitamin K (phylloquinone)	µg	2.1
**Lipids**		
Fatty acids, total saturated	g	0.038
14:00	g	0.001
16:00	g	0.027
18:00	g	0.009
Fatty acids, total monounsaturated	g	0.047
16:1 undifferentiated	g	0.001
18:1 undifferentiated	g	0.047
Fatty acids, total polyunsaturated	g	0.052
18:2 undifferentiated	g	0.027
18:3 undifferentiated	g	0.026
Phytosterols	mg	12
**Amino Acids**		
Tryptophan	g	0.009
Threonine	g	0.022
Isoleucine	g	0.02
Leucine	g	0.03
Lysine	g	0.032
Methionine	g	0.01
Cystine	g	0.01
Phenylalanine	g	0.024
Tyrosine	g	0.014
Valine	g	0.024
Arginine	g	0.018
Histidine	g	0.015
Alanine	g	0.026
Aspartic acid	g	0.569
Glutamic acid	g	0.083
Glycine	g	0.023
Proline	g	0.039
Serine	g	0.03

USDA National Nutrient Database for Standard Reference, software version 3.9.2, 2018.

Supplementary Table S2.
Polyphenols present in raw Sweet Cherry (*Prunus avium* L.)

Polyphenol Type	Unit	Value per 100 g
Total polyphenols	mg	174.90
**Flavonoids**		
**Anthocyanins**		
Cyanidin 3-O-glucoside	mg	18.73
Cyanidin 3-O-rutinoside	mg	143.27
Pelargonidin 3-O-rutinoside	mg	1.24
Peonidin 3-O-glucoside	mg	0.76
Peonidin 3-O-rutinoside	mg	7.42
**Flavanols**		
(+)-Catechin	mg	1.5
(-)-Epicatechin	mg	7.78
(-)-Epicatechin 3-O-gallate	mg	0.09
(-)-Epigallocatechin	mg	0.05
Procyanidin dimer B1	mg	0.23
Procyanidin dimer B2	mg	2.1
Procyanidin dimer B3	mg	0.08
Procyanidin dimer B4	mg	0.18
Procyanidin dimer B5	mg	0.2
Procyanidin dimer B7	mg	1.01
Procyanidin trimer C1	mg	1.85
**Phenolic acids**		
**Hydroxycinnamic acids**		
3-Caffeoylquinic acid	mg	44.71
3-Feruloylquinic acid	mg	0.43
3-p-Coumaroylquinic acid	mg	38.43
4-Caffeoylquinic acid	mg	0.77
4-p-Coumaroylquinic acid	mg	1.27
5-Caffeoylquinic acid	mg	2.2

Phenol-Explorer, database on polyphenol content in foods, software version 3.6, 2015.

Supplementary Table S3.
Nutrients present in raw (black) Grape (*Vitis vinifera* L.)

Nutrient	Unit	Value per 100 g
**Proximates**		
Water	g	80.54
Energy	kcal	69
Protein	g	0.72
Total lipid (fat)	g	0.16
Carbohydrate, by difference	g	18.1
Fiber, total dietary	g	0.9
Sugars, total	g	15.48
**Minerals**		
Calcium, Ca	mg	10
Iron, Fe	mg	0.36
Magnesium, Mg	mg	7
Phosphorus, P	mg	20
Potassium, K	mg	191
Sodium, Na	mg	2
Zinc, Zn	mg	0.07
**Vitamins**		
Vitamin C, total ascorbic acid	mg	3.2
Thiamin	mg	0.069
Riboflavin	mg	0.07
Niacin	mg	0.188
Vitamin B-6	mg	0.086
Folate, DFE	µg	2
Vitamin A,	µg	3
Vitamin E (alpha-tocopherol)	mg	0.19
Vitamin K (phylloquinone)	µg	14.6
**Lipids**		
Fatty acids, total saturated	g	0.054
Fatty acids, total monounsaturated	g	0.007
Fatty acids, total polyunsaturated	g	0.048

USDA National Nutrient Database for Standard Reference, software version 3.9.2 2018-04-30

Supplementary Table S4.
Polyphenols present in raw (black) Grape (*Vitis vinifera* L.)

Polyphenols	Unit	Value per 100 g
Total polyphenols	mg	174.90
**Flavonoids**		
**Anthocyanins**		
Cyanidin 3-O-(6"-p-coumaroyl-glucoside)	mg	0.1
Cyanidin 3-O-glucoside	mg	1.08
Delphinidin 3-O-(6"-acetyl-glucoside)	mg	0.54
Delphinidin 3-O-glucoside	mg	2.63
Malvidin 3-O-(6"-acetyl-glucoside)	mg	9.66
Malvidin 3-O-(6"-p-coumaroyl-glucoside)	mg	9.91
Malvidin 3-O-glucoside	mg	39.23
Peonidin 3-O-(6"-p-coumaroyl-glucoside)	mg	0.34
Peonidin 3-O-glucoside	mg	5.8
Petunidin 3-O-(6"-p-coumaroyl-glucoside)	mg	0.05
Petunidin 3-O-glucoside	mg	2.76
**Flavanols**		
(+)-Catechin	mg	5.46
(-)-Epicatechin	mg	5.24
(-)-Epicatechin 3-O-gallate	mg	1.68
(-)-Epigallocatechin	mg	0.03
Procyanidin dimer B1	mg	0.43
Procyanidin dimer B2	mg	0.36
Procyanidin dimer B3	mg	0.12
Procyanidin dimer B4	mg	0.33
Procyanidin trimer C1	mg	0.38
**Flavonols**		
Quercetin 3-O-galactoside	mg	0.93
Quercetin 3-O-glucuronide	mg	2.15
**Phenolic acids**		
**Hydroxycinnamic acids**		
Caffeoyl tartaric acid	mg	1.13
p-Coumaroyl tartaric acid	mg	0.56
**Stilbenes**		
**Stilbenes**		
Piceatannol	mg	5.20E-03
Resveratrol	mg	0.15
Resveratrol 3-O-glucoside	mg	0.03
Trans-Resveratrol	mg	0.15
Trans-Resveratrol 3-O-glucoside	mg	1.00E-02

Phenol-Explorer, database on polyphenol content in foods, software version 3.6, 2015.

Supplementary Table S5.
Primer sequences used in qPCR amplification

Gene	Sequence	Product size (bp)	GenBank accession no/reference
Obrb	Forward 5’-CCAGTACCCAGAGCCAAAGT<br>Reverse 5’-GGATCGGGCTTCACAACAAGC	122	NM_012596.1
Socs3	Forward 5’-CTGGACCCATTCGGGAGTTC<br>Reverse n’-CTGGGAGCTACCGACCATTG	148	NM_053565.1
Ptp1b	Forward 5’-CCCTTTTGACCACAGTCGGA<br>Reverse 5’-TTGGTAAAGGGCCCTGGGTG	119	NM_012637.2
Pomc	Forward 5’-GAGGCGACGGAGGAGAAAAG<br>Reverse 5’-TGAGGCTCTGTCGCGGAAA	98	NM_139326.2
AgRP	Forward 5’-GAGAACTCTGGGAACAGGGC<br>Reverse 5’-CAAGCAAAGGCCATGCTGAC	140	NM_033650.1
Npy	Forward 5’-CTATCCCTGCTCGTGTGTTTGG<br>Reverse 5’-TGGTGATGAGATTGATGTAGTGTCG	136	Sun B et al. 2014
Mc4r	Forward 5’-CAACTCCTTTGCAAGCTCCG<br>Reverse 5’-TCCAACCTCCTAGGTCAGGG	129	NM_013099.3
Npy1r	Forward 5’-TCTTCTCTGCCCTTCGTGATC<br>Reverse 5’-TGAACGCCGCAAGTGATACA	73	NM_001113357.1
Ppia	Forward 5’-CTTCGAGCTGTTTGCAGACAA<br>Reverse 5’-AAGTCACCACCCTGGCACATG	138	NM_017101.1
Rplp0	Forward 5’-GGACCTCACCGAGATTAGGG<br>Reverse 5’-CCCACCTTGTCTCCAGTCTT	225	NM_022402.2

Obrb, leptin receptor isoform b; Socs3, suppressor of cytokine signaling 3; Ptp1b, protein tyrosine phosphate 1B; Pomc, proopiomelanocortin; Agrp, agouti-related protein; Npy, neuropeptide Y; Mc4r, melanocortin 4 receptor; Npy1r, neuropeptide Y receptor Y1; Ppia, peptidylprolyl isomerase A; Rplp0, ribosomal protein lateral stalk subunit P0.

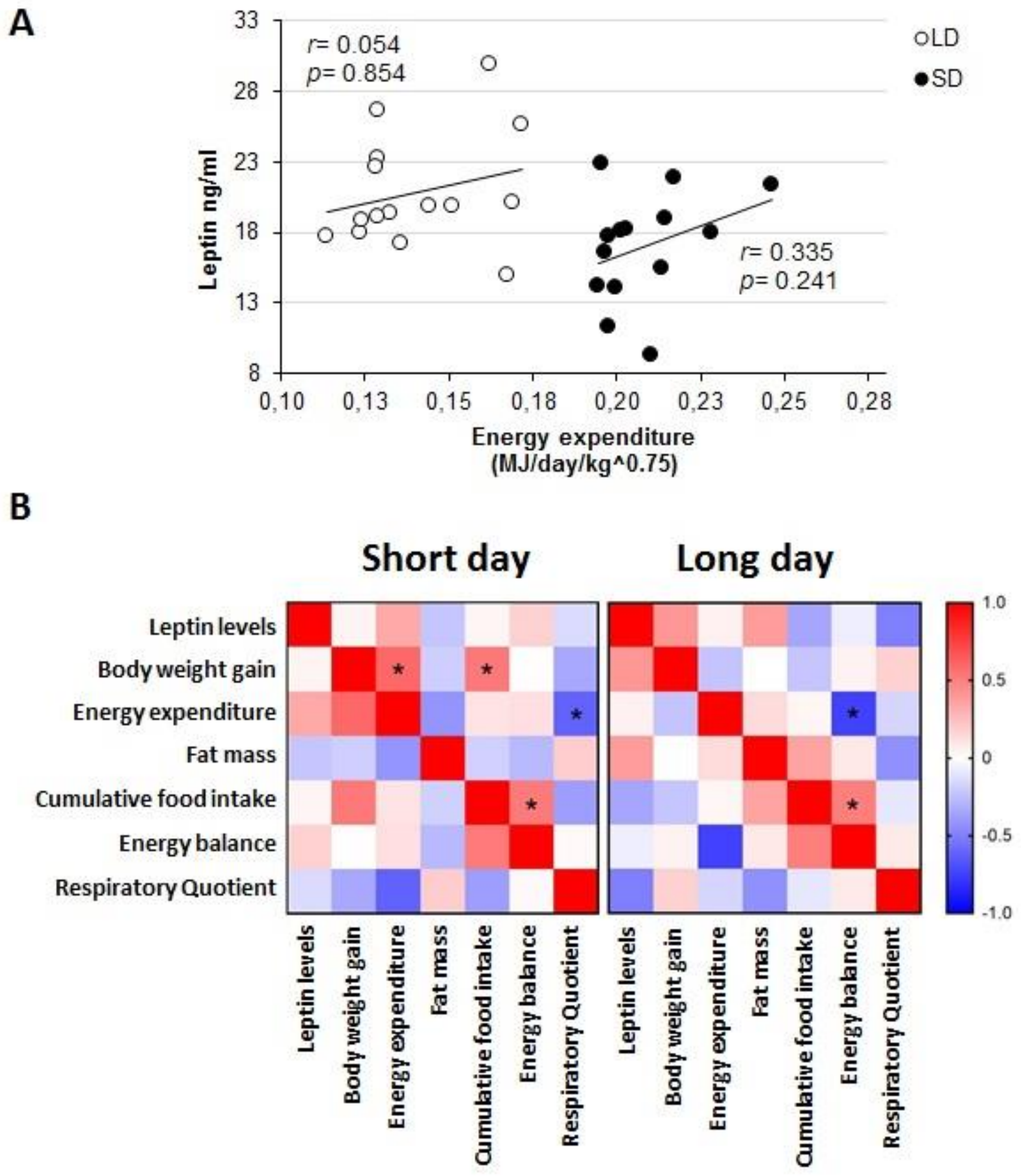

Supplementary Figure S1. Correlation analyses of metabolic variables in <u>non-obese animals</u>. (A) Global correlation plot between 24 h energy expenditure and circulating leptin levels of all non-obese animals in relation to either long-day (LD) or short-day (SD). **(B)** Heatmaps showing the Pearson correlation (r) coefficients between serum leptin (ng/ml), body weight gain (g), 24 h energy expenditure (MJ/day/kg^0.75), fat mass %, cumulative food intake (MJ), 24 h energy balance (MJ) estimated by the difference between energy intake and energy expenditure and finally the 24 h respiratory quotient in relation to either long-day (LD) or short-day (SD). Strengh and direction of the association between two variables is represented by *r* values ranging from -1 to 0 for negatively associated and 0 to 1 for positively associated parameters, coloured in blue to red respectively. Values are presented as the mean ± SEM of six animals per group. *adjusted P-value ≤0.05 taking the variables on the Y axis as reference.

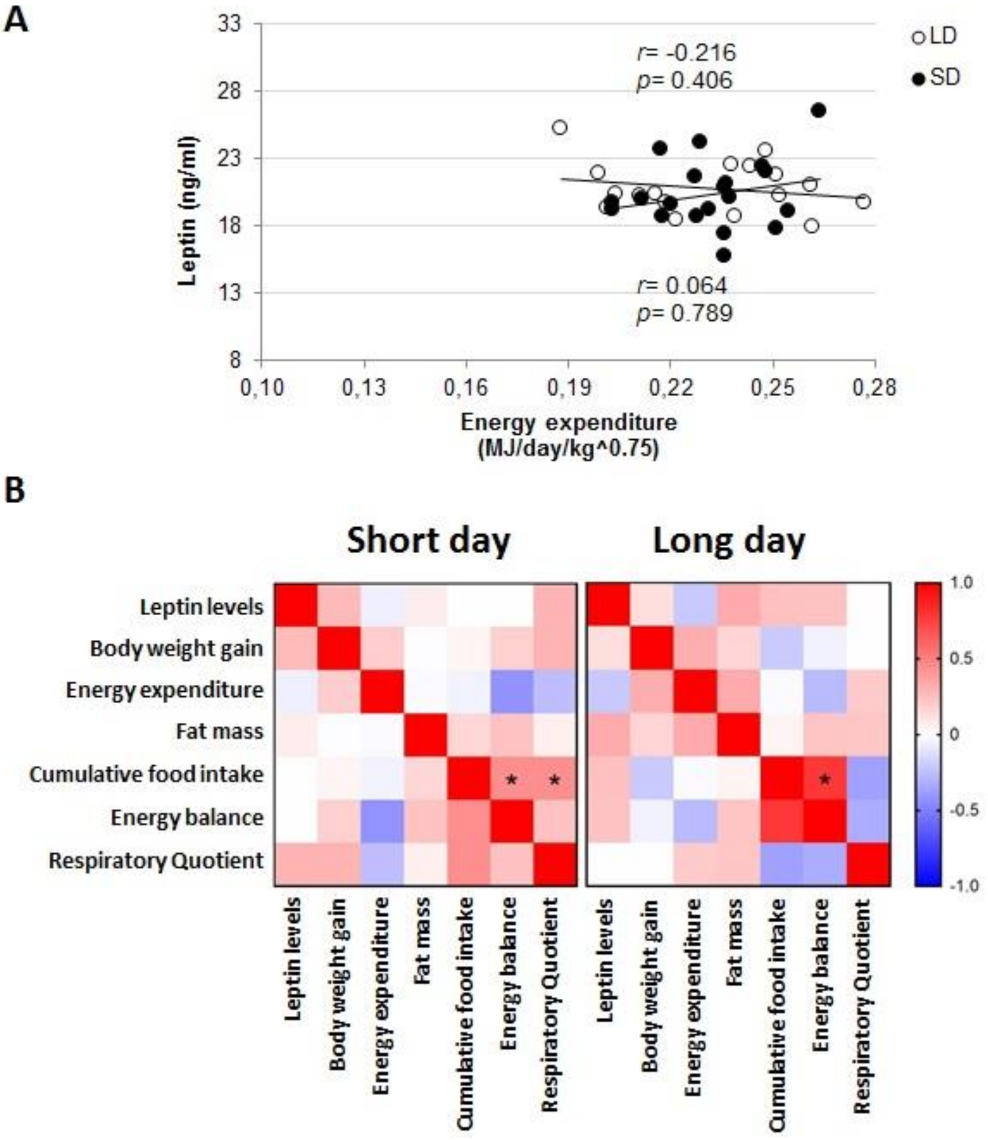

Supplementary Figure S2. Correlation analyses of metabolic variables in <u>obese animals</u>. (A) Global correlation plot between 24 h energy expenditure and circulating leptin levels of all obese animals in relation to either long-day (LD) or short-day (SD). **(B)** Heatmaps showing the Pearson correlation (r) coefficients between serum leptin (ng/ml), body weight gain (g), 24 h energy expenditure (MJ/day/kg^0.75), fat mass %, cumulative food intake (MJ), 24 h energy balance (MJ) estimated by the difference between energy intake and energy expenditure and finally the 24 h respiratory quotient in relation to either long-day (LD) or short-day (SD). Strengh and direction of the association between two variables is represented by *r* values ranging from -1 to 0 for negatively associated and 0 to 1 for positively associated parameters, coloured in blue to red respectively. Values are presented as the mean ± SEM of six animals per group. *adjusted P-value ≤0.05 taking the variables on the Y axis as reference.
